# A century-long monitoring of rice plant height during growth period in central Taiwan

**DOI:** 10.1038/s41597-025-05095-5

**Published:** 2025-05-05

**Authors:** Hungyen Chen, Yi-Chien Wu, Chih-Yung Teng, Cheng-Hong Li

**Affiliations:** 1 Department of Agronomy, National Taiwan University, Taipei, 106319 Taiwan; 2 Taichung District Agricultural Research and Extension Station, Ministry of Agriculture, Changhua, 515008 Taiwan

**Keywords:** Agroecology, Agroecology

## Abstract

The temporal variations in the crop traits of rice, one of the major grain crops, are garnering increasing attention from agronomist, ecologists and conservation biologists. However, long-term datasets of rice plant height during growth period with consistent cropping method at the consistent research field are rather rare. Here we describe a dataset of rice plant height containing 24446 records for 20 rice cultivars collected from a research farm in central Taiwan between 1925 and 2024, spanning a century. The plant heights were collected during the growth period for two cropping seasons (cool and warm) a year. Taiwan has good-quality cultivars and climatic conditions suitable for rice growth, making it an ideal place to study rice growth. Our data can be used to understand the rice growth cycle, investigate the temporal changes of rice plant height, construct the crop growth model, and to understand temporal variations in rice plant height concerning climate change, environmental factors, and anthropogenic pressures among cultivars and cropping seasons.

## Background & Summary

Rice plant height is not only an important component of crop canopy structure, but also a crucial way to increase crop yield. The increase in plant height represents an increase in aboveground biomass accumulation, which contributes to yield improvement^1,2^. Rice plant height also affects the severity of pests and diseases^3^; nevertheless, excessive plant height can cause plant lodging during the later stages of plant growth. Therefore, maintaining rice stalk strength within a certain range during growth period is necessary for increasing yield^2,4^. However, long-term datasets of rice plant height during growth period with consistent cropping method at the consistent research field are rather rare.

In Taiwan, rice cultivation includes indica and japonica type. Indica rice is suitable for tropical regions, with plants being heat-tolerant, having strong tillering ability, and producing slender grains with hard and non-sticky texture. On the other hand, japonica rice is more suitable for temperate environments, with plants being less heat-tolerant, having poorer tillering ability, and producing short and round grains with soft and sticky texture^5^. Rice cultivation in Taiwan has a history of more than 100 years, and the history of rice production can be traced back to before the Japanese colonial era. At that time, indica rice varieties were predominantly grown, and early indica type such as Shuangchiang (SC) was introduced during the Qing Dynasty. It was during the Japanese colonial era that the improvement of indigenous indica rice varieties began, along with the introduction of foreign varieties and the development of new varieties in Taiwan^6^. Around 1934, Taiwan primarily cultivated varieties like Wuchien (WC) and Paimifen (PMF) for the spring crops, and varieties like Paiko (PK), Chingkao (CK), and Niaoyao (NY) for the second crops. Some local farmers also introduced Hsiehlo (HL) varieties from Thailand^6^. In 1949, hybrid breeding of indica rice started in Taiwan, and varieties such as Taichung Native 1 (TN1) were developed by crossing Tichueh-Wuchien with Tsai-Yuan-Chung^6^. Starting from the 1970s, the main indica type rice varieties shifted from TN1 which was more suitable for processing, to Taichung Sen 3 (TCS3) and Taichung Sen 10 (TCS10) which were primarily used for rice consumption. Japonica type rice varieties were introduced and cultivated during the Japanese colonial era. Initially, the introduced Japanese varieties were not well-adapted to the cultivation environment in Taiwan, resulting in stunted plant growth, early flowering, increased susceptibility to diseases, and low yield^7^. For example, the Nakamura (NM) variety was severely affected by rice blast disease. Therefore, from 1926 to 1930, the agricultural research and extension stations selected dozens of disease-resistant varieties for farmers to plant, including Taichung Special 2 (TCSpe2) and Taichung Special 6 (TCSpe6)^7^. The agricultural improvement stations continued to conduct variety improvement and introduced hybrid breeding techniques to develop varieties with strong disease resistance, high yield, adaptability, and suitable rice quality for the Japanese taste. This led to the development of varieties such as Taichung 65 (TC65) and Taichung 150 (TC150), which were widely cultivated^6,7^. In the 1980s, Tainung 67 (TNG67) became a major japonica rice variety and Taichung 189 (TC189) was one of the top three most cultivated japonica rice varieties^6,7^. In the 2000s, varieties like Taikeng 8 (TK8) and Taikeng 9 (TK9) replaced TNG67 and became the main cultivated varieties^6^.

Although most early varieties are no longer suitable for cultivation in today’s environment, they still have preservation and research value. Therefore, studies on the rice plant height during growth period can provide a better understanding of the growth cycle and characteristics of these early rice cultivars, contributing to the enhancement of related information on cultivar traits. The cultivars of japonica rice in Taiwan, Pon-Lai rice, are the only rice varieties with good quality that can grow in areas with subtropical climates, relatively high temperatures and short sunshine, and has high yields^8,9^. Taiwan has high-quality cultivars and climatic conditions suitable for rice growth, making it an ideal place to study rice growth^9–13^.

Here we describe the long-term time series dataset of monitoring of rice plant height during growth period in central Taiwan between 1925 and 2024. Our data can be used to understand the temporal changes of rice plant height during growth period in central Taiwan, as well as differences of growth cycle among cultivars^14,15^. Additionally, these data can be used to construct the crop growth model of rice^16^. Finally, researchers can also use the data to understand temporal variations in rice plant height concerning climate change, environmental factors, and anthropogenic pressures^11–13^.

## Methods

A field experiment on rice growth was conducted between 1925 and 2024 at the research farm in the Taichung District Agricultural Research and Extension Station, Ministry of Agriculture, Executive Yuan, Taiwan (1925–1984: 24°09′N 120°41′E (WGS84), altitude 77 m above mean sea level; 1996–2024: 24°00′N 120°32′E, altitude 19 m above mean sea level). In 1984, the research station and farm were moved to a location approximately 20 km from the original location. The field site was located on the coastal plain of western Taiwan, where the soil is covered with alluvial material from the central mountain range, which is the principal mountain range on the Taiwan island. The parent materials of the soil were limestone, mudstone, and clay slate. The surface soil was light yellow in color and fertile for cultivation with a pH of 7.43. The soil quality has not changed over the years. Data of the research farm were previously described in Chen *et al*.^9,10^.

**Fig. 1:**
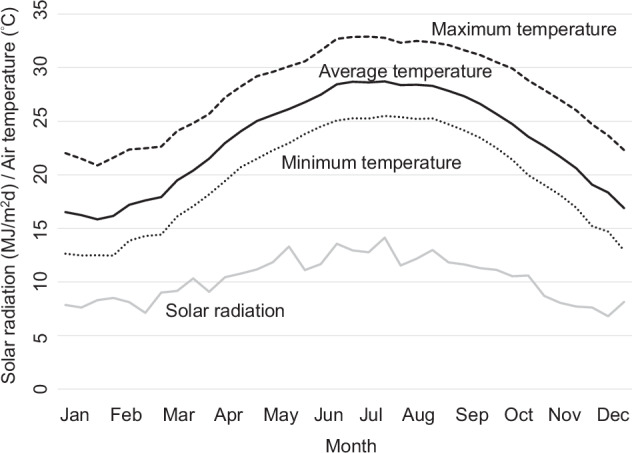
Mean values of the seasonal variations on the ten days averages of daily average, maximum, and minimum temperatures, and solar radiation in the research farm during 1990–2012.

The research was conducted across two cropping seasons (cool and warm) and the crop yields were measured by multiple researchers at the research station. The measurement methods and standards were kept consistent across different time periods throughout the experimental period. Consistency of data recording was ensured by detailed measurement and recording procedures established in the Taichung District Agricultural Research and Extension Station, a rigorous and well- managed government research unit. Seeds of the cool cropping season rice were sown in mid-January, and seedlings were dibbled either in late February or early March. The cool cropping season rice was harvested in either late June or early July. On the other hand, seeds of the warm cropping season rice were sown at the end of June, and seedlings were dibbled either in late July or early August. The warm cropping season rice was harvested in either late October or early November. Thus, the average temperature increases and decreases during the cool and warm farming seasons, respectively. The climate characteristics, i.e., daily average, maximum, and minimum temperature, and solar radiation in the research farm are shown in Fig. 1.

Rice seeds were initially grown in a nursery box, following which, sprouted seedlings were transplanted into the field by hand dibbling. The area of planting plot for each cultivar was 9 m^2^ (3 m × 3 m). During the period of 2005–2024, 4 plots were randomly selected and used for each cultivar; between 1999–2004, 3 plots were randomly selected and used for each cultivar; before 1999, there was a lack of data on the plot. Data of the experimental design were previously described in Chen *et al*.^9,10^. Four to six seedlings were dibbled in each hole; the rows were 30 cm wide and arranged in 15 cm intervals. A base fertilizer, with a nitrogen:phosphorus:potassium ratio (N:P:K) of 12:7.87:9.96, was added to the soil at a rate of 200 kg ha^−1^ 12 days before transplanting. The top dressing of fertilizer application was performed three times at 10–15, 20–30, and 55–65 days and at 7–10, 14–21, and 45–55 days after transplanting in the cool and warm cropping seasons, respectively. The top three fertilizers were added to the soil at a rate of 200 (N:P:K = 21:0:0), 200 (N:P:K = 12:5.68:10.79), and 150 kg ha^−1^ (N:P:K = 12:5.68:10.79) during both cropping seasons. Herbicides were applied during the cropping seasons, and insecticides were applied after checking the rice for symptoms of pests and diseases in rice. Data of the agricultural practices were previously described in Chen *et al*.^9,10^.

During the seedling stage, 20 seedlings were randomly selected from the nursery box for investigation, and the seedling heights of each cultivar were recorded. The plant heights of each cultivar were investigated during the young ear formation stage, heading stage, and maturity period. During the period of 2016–2024, 10 plants were randomly selected for each plot; between 2006–2013, 9 plants were randomly selected for each plot; before 2006, 12 plants were randomly selected for each plot for investigation. Before 1999, there was a lack of data on the seedling stage, and the plant height investigated in other stages were only average values. The growing days after seedling transplantation of rice were calculated based on different years, cropping seasons, transplanting dates and trait investigation dates at each growth period.

In total, 20 rice cultivars were used throughout the experimental period: Chingkao (CK), Hsiehlo (HL), Nakamura (NM), Niaoyao (NY), Paiko (PK), Paimifen (PMF), Shuangchiang (SC), Taichung 150 (TC150), Taichung 189 (TC189), Taichung 65 (TC65), Taichung Sen 10 (TCS10), Taichung Sen 2 (TCS2), Taichung Sen 3 (TCS3), Taichung Special 2 (TCSpe2), Taichung Special 6 (TCSpe6), Taikeng 9 (TK9), Taichung Native 1 (TN1), Tainan 11 (TN11), Tainung 67 (TNG67), and Wuchien (WC). Data of rice cultivars were previously described in Chen *et al*.^9,10^.

## Data Records

The dataset contains 24446 rice plant height records collected from the research farms of the Taichung District Agricultural Research and Extension Station. The data are presented in the form of a list of cropping seasons, recording dates (year, month, and day), growing days after seedling transplantation, cultivars (name and type), planting plots and plant heights^17^. The data contain 24446 rows (records) and 10 columns (cropping season, year, month, day, growing days after seedling transplantation, cultivar, cultivar type, plot, plant height, and plant height value type). The data are provided in a CSV file.

## Technical Validation

**Fig. 2:**
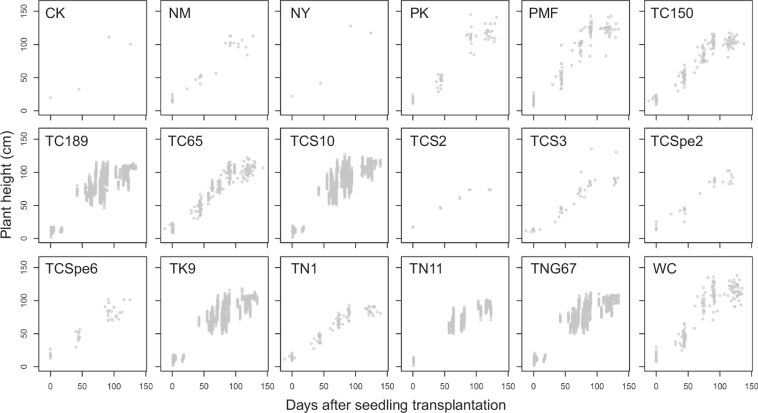
Scatter plots of plant height and growing days of the cultivars used during cool cropping season. Cultivars: Chingkao (CK), Nakamura (NM), Niaoyao (NY), Paiko (PK), Paimifen (PMF), Taichung 150 (TC150), Taichung 189 (TC189), Taichung 65 (TC65), Taichung Sen 10 (TCS10), Taichung Sen 2 (TCS2), Taichung Sen 3 (TCS3), Taichung Special 2 (TCSpe2), Taichung Special 6 (TCSpe6), Taikeng 9 (TK9), Taichung Native 1 (TN1), Tainan 11 (TN11), Tainung 67 (TNG67), and Wuchien (WC).

**Fig. 3:**
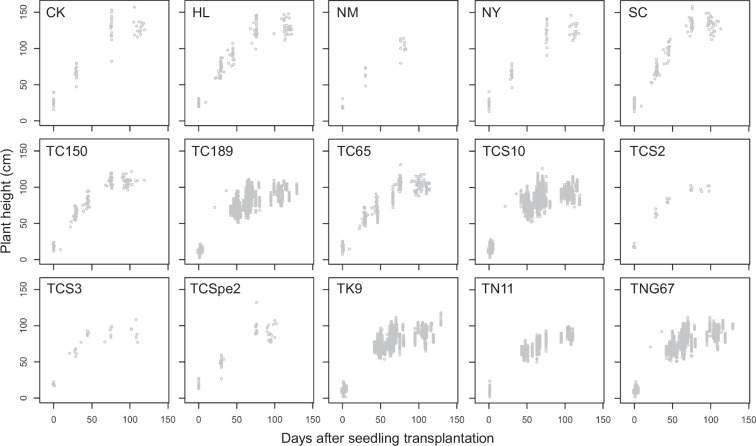
Scatter plots of plant height and growing days of the cultivars used during warm cropping season. Cultivars: Chingkao (CK), Hsiehlo (HL), Nakamura (NM), Niaoyao (NY), Shuangchiang (SC), Taichung 150 (TC150), Taichung 189 (TC189), Taichung 65 (TC65), Taichung Sen 10 (TCS10), Taichung Sen 2 (TCS2), Taichung Sen 3 (TCS3), Taichung Special 2 (TCSpe2), Taikeng 9 (TK9), Tainan 11 (TN11), and Tainung 67 (TNG67).

We implemented several validation procedures to maintain data quality. To demonstrate the value of the data, the relationship between growing days after seedling transplantation and rice plant height was investigated. The rice plant heights and the corresponding growing days after seedling transplantation of the cultivars used during cool and warm cropping seasons are shown in Figs. 2, 3. After constructing the dataset, we checked for potential outliers and NA and carefully summarized the information of cultivars and recording dates. The duration, number of years, and number of records of the cultivars used in the experiments during both cropping seasons are shown in Table 1. We defined an observation of plant height to be an outlier if it is 1.5 times the interquartile range greater than the third quartile or 1.5 times the interquartile range less than the first quartile among the observations randomly selected at the same time. To minimize inconsistencies and potential errors in the data entry, we carefully summarized the information of cultivars and recording dates, and corrected the possible data mistyping.

**Table 1:** Duration, number of years, and number of records of the cultivars used during cool and warm cropping seasons.

Cultivar	Cool cropping season			Warm cropping season		
	Duration	Number of years	Number of records	Duration	Number of years	Number of records
CK	1932	1	4	1926–1942	16	62
HL	NA	NA	NA	1943–1976	30	136
NM	1925–1931	7	28	1926–1931	6	23
NY	1932	1	4	1926–1942	16	62
PK	1925–1944	18	70	NA	NA	NA
PMF	1945–1976	28	131	NA	NA	NA
SC	NA	NA	NA	1943–1976	30	136
TC150	1945–1984	36	171	1945–1983	35	165
TC189	1996–2024	26	2583	1984–2023	25	2436
TC65	1930–2021	51	365	1930–2021	50	358
TCS10	1996–2024	26	1883	1984–2023	25	2436
TCS2	1979–1980	2	10	1977–1983	7	35
TCS3	1974–1984	9	45	1977–1984	8	40
TCSpe2	1925–1932	8	32	1926–1944	19	71
TCSpe6	1933–1944	11	42	NA	NA	NA
TK9	1999–2024	23	2580	1999–2023	21	2428
TN1	1964–1984	19	95	NA	NA	NA
TN11	2017–2024	8	1120	2016–2023	7	980
TNG67	1996–2024	26	2583	1984–2023	25	1736
WC	1925–1976	45	196	NA	NA	NA

Cultivars: Chingkao (CK) Hsiehlo (HL), Nakamura (NM), Niaoyao (NY), Paiko (PK), Paimifen (PMF), Shuangchiang (SC), Taichung 150 (TC150), Taichung 189 (TC189), Taichung 65 (TC65), Taichung Sen 10 (TCS10), Taichung Sen 2 (TCS2), Taichung Sen 3 (TCS3), Taichung Special 2 (TCSpe2), Taichung Special 6 (TCSpe6), Taikeng 9 (TK9), Taichung Native 1 (TN1), Tainan 11 (TN11), Tainung 67 (TNG67), and Wuchien (WC).

The varying environmental conditions across different years might have influenced the observed rice plant height. The annual trend of air temperature, rainfall, and sunshine duration recorded in the research farm of the Taichung District Agricultural Research and Extension Station can be found in Fig. 1 in Chen *et al*.^11^. Using the data obtained from the same experiment, Chen *et al*.^11^ estimated the relationships between rice yield and the climate variables using the time series of their first difference values. They computed the total relative and annual actual yield changes using regression coefficients for each climate variable to reveal the impacts of climate change on yields and the associated temporal variations during the overall experimental period. In another study, Chen *et al*.^12^ analyzed the same data of rice growth to evaluate the effects of water-deficit stress induced by climate change on the rice yields.

## Data Availability

No custom code was used to generate or process the data described in this manuscript.
